# Editorial: Omics techniques to optimize plant-microbe interactions under climate change

**DOI:** 10.3389/fpls.2024.1423421

**Published:** 2024-06-14

**Authors:** Carmen Bianco, Dilfuza Egamberdieva, Raffaella Balestrini, Francesca Taranto

**Affiliations:** ^1^ Instutute of Biosciences and BioResources, National Research Council, Naples, Italy; ^2^ Faculty of Biology, National University of Uzbekistan, Tashkent, Uzbekistan; ^3^ Institute of Fundamental and Applied Research, National Research University Tashkent Institute of Irrigation and Agricultural Mechanization Engineers (TIIAME), Tashkent, Uzbekistan

**Keywords:** environmental stresses, plant growth-promoting bacteria, nitrogen-fixing rhizobia, arbuscular mycorrhizal fungi, fungal and bacterial endophytes, omics technologies

The new climatic scenario along with the constant increase in the world population requires a great effort for boosting crop yield and quality.

Environmental stresses affect not only plant growth and yield, but also the composition and functionality of microbial communities interacting with plants, including beneficial ones.

Addressing the gap in knowledge related to plant-microbe interactions and crops genotype to phenotype relationships could contribute to the development of innovative strategies aimed at improving plant survival under environmental stress conditions (Mukhtar et al., 2023). The latest advances in omics and multi-omics approaches could be exploited to fill that gap (Kimotho and Solomon, 2024).

This Frontiers Research topic gathers different contributions addressing the use of integrated systems to study beneficial interactions between plants and soil bacteria (including mutualists, such as arbuscular mycorrhizal fungi and rhizobia, and commensals, such as plant growth-promoting bacteria, fungal and bacterial endophytes), and discussing the application of different agronomic practices to improve plant growth and productivity under various climatic conditions.

Two reviews address the use of biostimulants based on microorganisms (such as bacteria, fungi and mycorrhizae) to improve plant growth, productivity and stress tolerance, and highlighted that several issues remain to be resolved.

The Review by Ma et al. deals with the underlying mechanisms of action mediated by diverse biostimulants in relation to abiotic stress mitigation, as well as discusses the current challenges in their commercialization and implementation in agriculture under changing climate conditions. Based on their recommendations, future advances in the application of biostimulants could lead to the development of sustainable strategies to increase plant tolerance against adverse environmental conditions. The Review by Sena et al. provides a summary and update on the omics technologies, such as genomics, transcriptomics, proteomics, and metabolomics, employed to elucidate the complex network of plant-microorganism interactions for improving crop production and resilience. Considering the case of endophytes, they state that to optimize their use in a unique vision of health, a deep knowledge of the biology, mode of action and main interactions of endophytes in the complex microorganism-plant-environment system. Both reviews suggest that the knowledge of the mechanisms underlying the plant-microbe interactions should be considered in depth before evaluating a potential application of biostimulants to promote sustainable plant growth. In addition, the approaches explored in these Reviews offer potential solutions for future advances in the application of biostimulants to increase plant tolerance against adverse environmental conditions, such as adequate regulations, policies and production technologies. The clarification of biological mechanisms of action, and the development of –omic specific application technologies will be pivotal for contrasting impacts of climate change.

The importance of agronomic and genetic approaches to improve crop yields under various soil and climate conditions is addressed also in the Review by Govindasamy et al.

In this Review the literature regarding nitrogen loss, variables affecting NUE, and agronomic and breeding approaches for enhancing NUE in various crops are discussed. The manuscript suggests that to improve NUE, under various soil and climate conditions, precise timing and placement of N fertilizer, site-specific nutrient management, conservation tillage, crop residue retention and cultivation of crops with high biomass content should be incorporated. The work highlights that to breed superior genotypes with high NUE and to address challenges of climate change, the identification of genes or QTLs governing NUE combined with genomic selection, speed breeding and biotechnological techniques could represent a valuable approach in future breeding programs.

An example of an efficient approach to enhance crop production, ensuring stable yields under changing climate conditions, is reported by Guizani et al., in which the relationship between drought tolerance and specific physio-molecular adaptations is considered for the selection of wheat genotypes. This study shows how different wheat genotypes respond to drought and highlighted characteristics usable for the selection of genotypes resistant to water stress. It suggests that measures such as osmotic potential, modulus of elasticity and stomatal conductance could be used as primary selection criteria for the robust screening of numerous genotypes in wheat breeding programs.

Soil and rhizosphere characteristics determine the structure and diversity of bacterial communities interacting with plants. Fan et al. examine the rhizosphere microbial populations associated with three distinct types of alfalfa grown in soils with different salinity levels. According to their findings, certain dominating rhizosphere bacteria are abundantly enriched, and this is a major factor in the high salt tolerance of plant varieties. Their findings provide evidence that salinity plays a pivotal role in shaping the composition and structure of alfalfa rhizosphere microbiota and that the abundance of relevant bacterial genera is influenced and regulated by plant genotype.

Environmental perturbations resulting from global changes can alter the phyllosphere by influencing the crosstalk between plants and their microbiomes. Wang et al. apply the high-throughput 16S rRNA gene amplicon sequencing techniques to examine the bacterial communities residing on the leaf surfaces of an oilseed tree, yellowhorn (*Xanthoceras sorbifolium*), grown at four distinct locations in China with varying climatic conditions. They found that the bacterial community composition differed significantly among the four sampled site regions, indicating the possible impact of climatological factors upon the phyllosphere microbiome.

The questions addressed in the present Research Topic suggest that multi-omics approaches are needed to delve deeper into the mechanisms underlying plant-microbe interactions and to fully exploit them. Indeed, the use of different -omic approaches at the same time could allow to analyze different aspects influencing this interaction and to obtain broader information.

The application of multi-omics approaches will need to consider that climate change can alter the composition and interactions between plant and soil microbial communities through a variety of mechanisms that may be mediated by plant genotype, microbes, and environmental factors.

We believe that readers of the journal will find this Research Topic fascinating and helpful, and that it will increase their understanding of plant-microbe interactions and the value of current multi-omics techniques. We extend our gratitude to each author who has contributed to this Research Topic. We also thank all the reviewers, managing editors, and editorial professionals that helped with the editing and article production.

## Author contributions

CB: Writing – original draft, Writing – review & editing. DE: Writing – original draft, Writing – review & editing. RB: Writing – review & editing. FT: Writing – review & editing.
